# A Report on the Use of Biparametric MRI in Prostate Cancer Diagnosis: A Two-Year Single Centre Experience

**DOI:** 10.7759/cureus.91630

**Published:** 2025-09-04

**Authors:** Quadri A Sanni, Sadiq Abu, Tolulope Ogunfowora, Abdulkadir Mohamed, James Tsejime, Khaled Bseikri, Adnan Malik, Amman Malik, Andrea Ginepri, Jane Anderson, Rahim Kaba, Maya Harris, Rajasekaran Siddhan, Ashraf Fahmy

**Affiliations:** 1 Urology, South Warwickshire University NHS Foundation Trust, Warwick, GBR; 2 General Surgery, South Warwickshire University NHS Foundation Trust, Warwick, GBR; 3 Surgery, Sandwell and West Birmingham NHS Trust, Birmingham, GBR; 4 Breast Surgery, Glenfield Hospital, Leicester, GBR

**Keywords:** biparametric mri, dynamic contrast enhancement, gadolinium, oncology, prostate cancer

## Abstract

Introduction: The use of pre-biopsy MRI scans is now a standard of care in the evaluation of men with elevated prostate-specific antigen (PSA); however, the use of biparametric MRI (bpMRI) has been a source of controversy amongst health professionals. The study aimed to analyse our experience with the use of bpMRI in evaluating suspected prostate cancer in conjunction with other clinical indicators, such as PSA density (PSAD) and family history of prostate cancer.

Methods: This retrospective, single-centre study consisted of 404 patients who had an MRI due to elevated PSA and subsequent prostate biopsies.

Result: The average age of the study population was 69.7±7.3 years, with the majority of the patients above 70 years. A statistically significant positive correlation was observed between PSAD and Prostate Imaging-Reporting and Data System (PI-RADS) scores (r=0.2, p<0.001). Higher PSAD groups showed an increased proportion of PI-RADS 4 and 5 lesions. There was a significant positive correlation (r=0.2, p<0.001) between PSAD and International Society of Urological Pathology (ISUP) grade, indicating that the detection of clinically significant and higher-grade prostate cancer increases with PSAD. High-risk prostate cancers (ISUP grades 4 and 5) were detected in the high-risk category of PSAD (14.6% and 18%, respectively). PI-RADS scores correlated significantly with ISUP grades (r=0.3, p<0.001).

Conclusion: Our study demonstrated that bpMRI-detected PI-RADS scores significantly correlated with PSAD, histological diagnosis (ISUP grade), and clinically significant prostate cancer detection. bpMRI has a role in the diagnostic value of prostate cancer when combined with PSAD and a family history of prostate cancer. It is effective in detecting clinically significant prostate cancers at a lower cost and shorter time while eliminating the potential harm of the use of gadolinium.

## Introduction

Prostate cancer is the second most common cause of cancer-related deaths in men [1]. Magnetic resonance imaging (MRI) of the prostate has become the standard of care in evaluating men with elevated prostate-specific antigen (PSA) before prostate biopsy [2-6]. To have a standard protocol, report, and interpretation, a Prostate Imaging-Reporting and Data System (PI-RADS) v2.1 was developed [7], and currently, multiparametric MRI (mpMRI) is used, which includes the following sequences: T2-weighted (T2W) and diffusion-weighted imaging (DWI), as well as administration of a contrast agent to acquire dynamic contrast-enhanced (DCE) MRI [7,8]. mpMRI is a detailed MRI scan of the prostate, which involves the use of contrast, and biparametric MRI (bpMRI) does not involve the use of contrast [9,10]. mpMRI takes a relatively long time, approximately 30 to 45 minutes [9]. In addition to being time-consuming, it comes at a higher cost and has potential side effects with contrast use [10,11]. bpMRI, on the other hand, is efficient and more cost-effective, has shorter study time, eliminates patient discomfort and the need for physician presence during the administration of contrast, and has potential negative health effects [10,11]. Consequently, DCE MRI has been shown to have a limited role in the current reporting system due to its drawbacks [9]. Due to increasing demand for diagnostic MRI in prostate cancer, a bpMRI has been a subject of consideration to aid the diagnostic pathway. It could be an alternative MRI for the detection of clinically significant prostate cancer (csPCa), and several studies have shown promising results [12-14]. We report our experience of the use of bpMRI in prostate cancer diagnostic pathways over two years in the setting of a district general hospital.

## Materials and methods

This retrospective, single-centre study included 558 patients referred for the evaluation of prostate cancer. Patients who were unable to undergo prostate MRI before biopsy or who had benign histology were excluded.

A total of 404 patients who underwent prostate MRI and subsequently proceeded to transperineal prostate biopsy were included. Biopsy decisions were made following a multidisciplinary team (MDT) review of MRI prostate, which incorporated prostate cancer risk assessment based on serum PSA, digital rectal examination (DRE), family history of prostate cancer, and PSA density (PSAD).

PSAD was calculated using the most recent PSA value and MRI-derived prostate volume. Patients were stratified into four risk groups based on PSAD [15]: (1) low risk: <0.1 ng/mL/cc, (2) low-intermediate risk: 0.1-0.15 ng/mL/cc, (3) high-intermediate risk: 0.15-0.2 ng/mL/cc, and (4) high risk: >0.2 ng/mL/cc.

A threshold of 0.15 ng/mL/cc was considered significant. PI-RADS scores were validated at MDT meetings by uroradiologists who reviewed MRI images and compared them with the reported PI-RADS scores. PI-RADS 2 lesions were generally considered clinically insignificant unless associated with a PSAD>0.2 ng/mL/cc, a strong family history of prostate cancer, or an abnormal DRE. A shared decision-making process with the patient was used to offer a prostate biopsy. Histopathological grading was performed using the International Society of Urological Pathology (ISUP) classification [16] based on the Gleason scores. Patients were risk-stratified into four groups: low, low-intermediate, high-intermediate, and high risk, based on a composite of PSAD, MRI PI-RADS score, and ISUP grade. ISUP grade 2 (Gleason 3+4) was considered to be csPCa.

The relationships between PI-RADS scores and ISUP grade, as well as between PSAD and PI-RADS scores, were assessed using Spearman’s correlation coefficient with a 95% confidence interval. A p-value<0.05 was considered statistically significant.

## Results

**Figure 1:**
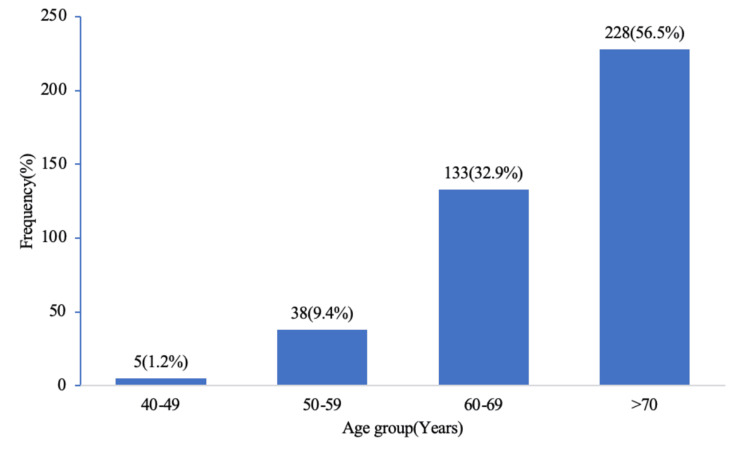
Age distribution with mean age (69.7±7.3 years) Data are presented as mean ± standard deviation or N (%).

**Figure 2:**
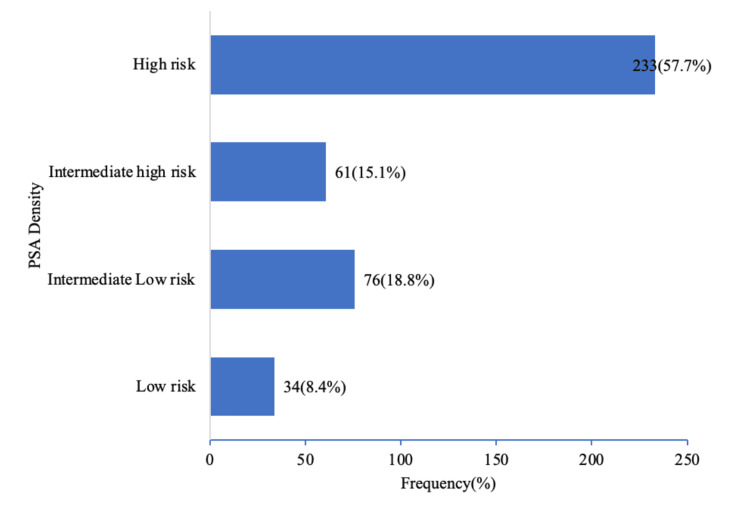
Bar graph showing PSAD distribution of participants The modal PSAD was in the high-risk group (233 (57.7%)). PSAD, Prostate-specific antigen density [15]

The age distribution of our study showed that the incidence of prostate cancer increases with age, with an average age of 69.7±7.3 years, as shown in Figure 1. The majority of the patients had PI-RADS 4, as revealed in Table 1 below, and high-risk PSAD was frequently encountered, as demonstrated below in Figure 2. 379 (93.7%) patients had csPca, with the highest prevalence in the low-intermediate risk group, 228 (56.4%), as shown in Table 2 below.

**Table 1:** PI-RADS scores The majority of patients (155 (38.4%)) had PI-RADS 4. PI-RADS, Prostate Imaging-Reporting and Data System [3]

PI-RADS scores	Frequency (N=404)	Percentage
2	34	8.4
3	115	28.5
4	155	38.4
5	100	24.8

**Table 2:** ISUP grade distribution of the patients The majority (228 (56.4%)) of patients were in the low-intermediate risk category. ISUP: International Society of Urologic Pathology [16]

ISUP grade	Frequency (N=404)	Percent
Low risk (3+3)	25	6.2
Low-intermediate risk (3+4)	228	56.4
High-intermediate risk (4+3)	49	12.1
High risk (4+4)	49	12.1
High risk (5+5)	53	13.1

A statistically significant but weak positive correlation was observed between PSAD and PI-RADS scores (r=0.2, p<0.001). Higher PSAD groups showed an increased proportion of PI-RADS 4 and 5 lesions. PI-RADS 5 was observed in 72 (32.6%) patients with high-risk PSAD. PI-RADS 2 and 3 scores were more frequent in lower PSAD groups. This is demonstrated in Table 3 below.

**Table 3:** Relationship between PSAD and PI-RADS scores A statistically significant but weak positive correlation was observed between PSAD and PI-RADS scores (r=0.2, p<0.001). *A p-value <0.05 was considered statistically significant. PI-RADS, Prostate Imaging-Reporting and Data System; PSAD, Prostate-specific antigen density [3,15]

	PSAD				r	p-value
	Low risk (n=34), n (%)	Low-intermediate risk (n=76), n (%)	High-intermediate risk (n=61), n (%)	High risk (n=233), n (%)		
PI-RADS scores					0.2	<0.001^*^
2	2 (5.9)	5 (6.5)	10 (16.4)	17 (7.3)		
3	12 (35.3)	29 (38.2)	18 (29.5)	56 (24.0)		
4	16 (47.1)	29 (38.2)	26 (42.6)	84 (36.1)		
5	4 (11.8)	13 (17.1)	7 (11.5)	76 (32.6)		

Table 4 below shows a positive significant correlation (r=0.2, p<0.001) between PSAD and ISUP grade, indicating that the detection of clinically significant and higher-grade prostate cancer increases with PSAD. High-risk prostate cancers (ISUP grade 4 and 5) were detected in the high-risk category of PSAD (34 (14.6.0%) and 42 (18%), respectively). ISUP 1 was associated with the low-risk category (5 (14.7%)). 

**Table 4:** Relationship between PSAD and ISUP scores There is a positive and significant correlation (r=0.2, p<0.001) between PSAD and ISUP grade, indicating that the detection of clinically significant and higher-grade prostate cancer increases with PSAD. Data are represented as n(%). *A p-value <0.05 was considered statistically significant. ISUP, International Society of Urological Pathology; PSAD: Prostate-specific antigen density [15,16]

	PSAD				r	p-value
	Low risk (n=34), n (%)	Low-intermediate risk (n=76), n (%)	High-Intermediate risk (n=61), n (%)	High risk (n=233), n (%)		
ISUP grade					0.2	<0.001^*^
Low risk (3+3)	5 (14.7)	6 (7.9)	4 (6.6)	10 (4.3)		
Low-intermediate risk (3+4)	20 (58.8)	48 (63.2)	42 (68.9)	118 (50.6)		
High-intermediate risk (4+3)	3 (8.8)	11 (14.5)	6 (9.8)	29 (12.4)		
High risk (4+4)	4 (11.8)	5 (6.5)	6 (9.8)	34 (14.6)		
High risk (5+5)	2 (5.9)	6 (7.9)	3 (4.9)	42 (18.0)		

There is a positive correlation between PI-RADS score and ISUP grades (r=0.3, p<0.001), as demonstrated in Table 5 below.

**Table 5:** A positive and significant correlation between PI-RADS scores and ISUP grades (r=0.3, p<0.001) Data are represented as n (%). *A p-value <0.05 was considered statistically significant. ISUP, International Society of Urological Pathology; PI-RADS, Prostate Imaging-Reporting and Data System [3,16]

Variable	PI-RADS scores				r	p-value
	2 (n=34), n (%)	3 (n=115), n (%)	4 (n=155), n (%)	5 (n=100), n (%)		
ISUP grade					0.3	<0.001^*^
Low risk (3+3)	5 (14.7)	10 (8.7)	8 (5.2)	2 (2.0)		
Low-intermediate risk (3+4)	22 (64.7)	74 (64.3)	96 (61.9)	36 (36.0)		
High-intermediate risk (4+3)	3 (8.8)	12 (10.4)	17 (11.0)	17 (17.0)		
High risk (4+4)	2 (5.9)	9 (7.9)	23 (14.8)	15 (15.0)		
High risk (5+5)	2 (5.9)	10 (8.7)	11 (7.1)	30 (30.0)		

For low-risk cancer (ISUP 1; Gleason 3+3), as the PI-RADS score increases, the proportion of low-risk cases decreases. In terms of the low-intermediate risk (ISUP 2: 3+4), this was the highest in PI-RADS 3 (74 (64.3%)) and PI-RADS 4 (96 (61.9%)) and started to decline in PI-RADS 5 (36 (36.0%)), suggesting progression to higher ISUP grades. The high-intermediate risk (ISUP 3: 4+3) showed an increasing trend, as with the PI-RADS score. Regarding the high risk (ISUP 4: 4+4 and ISUP 5: 5+5), PI-RADS 5 has the highest proportion of ISUP 5 (30 (30.0%)) and ISUP 4 (15 (15.0%)).

## Discussion

In this study, as shown in Figure 1, the mean age of the study population was 69.7±7.3 years, with the majority of the patients above 70 years. This is consistent with the findings that prostate cancer increases with age [17-19]. 

Patients included in this study predominantly had PI-RADS scores of 3, 4, and 5, with proportions of 115 (28.5%), 155 (38.4%), and 100 (24.8%), respectively, as shown in Table 1. Only a small number of patients, 34 (8.4%), with a PI-RADS score of 2, were included in this study. Though considered to be a negative MRI in the current guidelines, consideration for biopsy is warranted in those patients with significant PSAD >0.2 ng/mL/cc [20]. The use of PSAD becomes important in cases where MRI results are equivocal, allowing for more informed and shared clinical decisions in proceeding with prostate biopsy. This study demonstrates a statistically significant association between PSAD and bpMRI PI-RADS score (r=0.2), as shown in Table 3. The higher PSAD was associated with a greater proportion of PI-RADS 4 and 5 lesions, which are predictive of csPCa. This supports previous studies suggesting that PSAD may enhance the diagnostic accuracy of mpMRI [21]. 

From Table 4, our findings demonstrate a statistically significant correlation between PSAD and ISUP grade, consistent with previous studies [22]. This finding highlights the potential of PSAD in risk-adapted decision-making and the established evidence that men with elevated PSAD and higher-grade tumours may benefit from early intervention, while those with lower PSAD and grade group 1 disease may be candidates for active surveillance [23]. As shown in Table 5, our findings demonstrated that PI-RADS score on bpMRI has a significant positive correlation with ISUP grade (r=0.3, p<0.001), indicating that higher bpMRI PI-RADS lesion biopsies are more likely to yield more aggressive prostate cancer. This is consistent with previous studies that support the predictive value of PI-RADS (mpMRI) in assessing tumour grade and potential clinical outcomes using mpMRI [24]. Twilt et al., in an observational multicentre, multinational study conducted with 400 mpMRI examinations from four European centres, demonstrate that the diagnostic capability of bpMRI in detecting csPCa was non-inferior to that of mpMRI in men without prior csPCa findings and prostate treatment [25]. In addition, our results support that bpMRI is similar in diagnostic performance to mpMRI, which is in consonance with many studies, including recent meta-analyses [10,26-28]. Furthermore, a head-to-head comparison in a systematic review and meta-analysis by Woo et al. showed that the performance of bpMRI was similar to that of mpMRI in the diagnosis of prostate cancer [29]. In a recent prospective, international, multicentre trial, prostate Imaging using MRI ± contrast enhancement (PRIME), assessing whether bpMRI is non-inferior to mpMRI in the diagnosis of csPCa, it was demonstrated that bpMRI detected csPCa at the same rate as mpMRI, confirming that bpMRI without contrast-enhanced (DCE) sequences is non-inferior to standard mpMRI for detection of csPCa [30]. bpMRI stands out as a faster, cheaper, and equally effective alternative for transforming prostate cancer diagnostics for better patient access, and it avoids contrast-related environmental harm and reduces medical waste by reducing CO_2_ emissions [30].

Limitations

Our study is a single-centre retrospective study, and there is no direct correlation with mpMRI. Interobserver variability in PI-RADS scoring is another limitation of our study. There is also a lack of follow-up data on whether those who were not biopsied subsequently developed significant prostate cancer.

## Conclusions

bpMRI, combining T2W imaging with DWI and omitting DCE sequences, is a promising, efficient tool for prostate cancer evaluation, and it offers some advantages such as shorter scan times, lower cost, and no gadolinium injection. Our study demonstrated that bpMRI-detected PI-RADS scores significantly correlated with PSAD, histological diagnosis (ISUP grade), and clinically significant prostate cancer detection. To achieve good results, the protocol must be standardized, of high quality, and reported by well-trained, experienced radiologists for accurate interpretation.
